# Global Thrombosis Test – a possible monitoring system for the effects and safety of dabigatran

**DOI:** 10.1186/s12959-015-0069-6

**Published:** 2015-12-08

**Authors:** Kazunori Otsui, Diana A. Gorog, Junichiro Yamamoto, Takayuki Yoshioka, Sachiyo Iwata, Atsushi Suzuki, Toru Ozawa, Asumi Takei, Nobutaka Inoue

**Affiliations:** Kobe Rosai Hospital, Kobe, Japan; Imperial College, London, UK; Kobe Gakuin University, Kobe, Japan; Department of Cardiovascular Medicine, Kobe Rosai Hospital, 4-1-23, Kagoike Touri, Chuo-Ku, Kobe, 651-0053 Japan

**Keywords:** Atrial fibrillation, Novel oral anticoagulants, Warfarin

## Abstract

**Background:**

Dabigatran is an alternative to warfarin (WF) for the thromboprophylaxis of stroke in patients with non-valvular atrial fibrillation (NVAF). The advantage of dabigatran over WF is that monitoring is not required; however, a method to monitor the effect and the safety of dabigatran is not currently available. The Global Thrombosis Test (GTT) is a novel method to assess both clot formation and lysis activities under physiological conditions.

**Objective:**

The aim of this study was to evaluate whether treatment with dabigatran might affect shear-induced thrombi (occlusion time [OT], sec) by the GTT, and to investigate the possibility that the GTT could be useful as a monitoring system for dabigatran.

**Patients/Methods:**

The study population consisted of 50 volunteers and 43 NVAF patients on WF therapy, who were subsequently switched to dabigatran. Using the GTT, the thrombotic status was assessed one day before and 1 month after switching anticoagulation from WF to dabigatran.

**Results:**

The OT was 524.9 ± 17.0 sec in volunteers whereas that of NVAF patients on WF therapy was 581.7 ± 26.3 sec. The switch from WF to dabigatran significantly prolonged OT (784.5 ± 19.3 sec). One patient on WF therapy and 12 patients on dabigatran therapy were shown to have OT > 900 sec.

**Conclusion:**

The GTT could be used to assess the risk of dabigatran-related bleeding complications.

## Introduction

Anticoagulation is recommended for the prevention of thrombotic events in the majority of patients with atrial fibrillation (AF). Warfarin is a well-established anticoagulant; however, there are some limitations including large inter-individual variability in dose–response and its narrow therapeutic window. Furthermore, treatment with warfarin requires routine analytical work-ups and visits to monitor prothrombin time-international normalized ratio (PT-INR); therefore this therapy may adversely affect health-related quality of life. Novel oral anticoagulants (NOAC), including dabigatran are alternatives to warfarin for preventing thromboembolic events in patients with non-valvular AF (NVAF). Recent clinical trials have demonstrated the non-inferiority of NOACs to warfarin in this setting [1, 2]. The major advantage of NOAC over warfarin is that routine laboratory monitoring is claimed to be dispensable. However, there are no clinical tools available to indicate the bioactivity of NOAC and the risk of major bleeding, similar to the PT-INR, as is the case with warfarin.

The Global Thrombosis Test (GTT) is a novel comprehensive test of platelet reactivity, coagulation (thrombin generation), and spontaneous (endogenous) thrombolytic activity. This *in vitro* assay system evaluates both high-shear-induced thrombotic reactions and subsequent thrombolysis under physiological conditions by using non-anticoagulated blood samples. The GTT evaluates the time required to form an occlusive thrombus (occlusion time [OT]), and the time to lyse this thrombus (lysis time [LT]) are evaluated by the GTT [3]. Recently, we reported the validity of GTT as a monitoring system [4]. The aim of this study was to evaluate whether treatment with dabigatran might affect the OT or LT assessed by the GTT, and to investigate the possibility that the GTT could be useful as a monitoring system to evaluate the effect of dabigatran.

## Methods

### Study Protocol

**Fig. 1:**
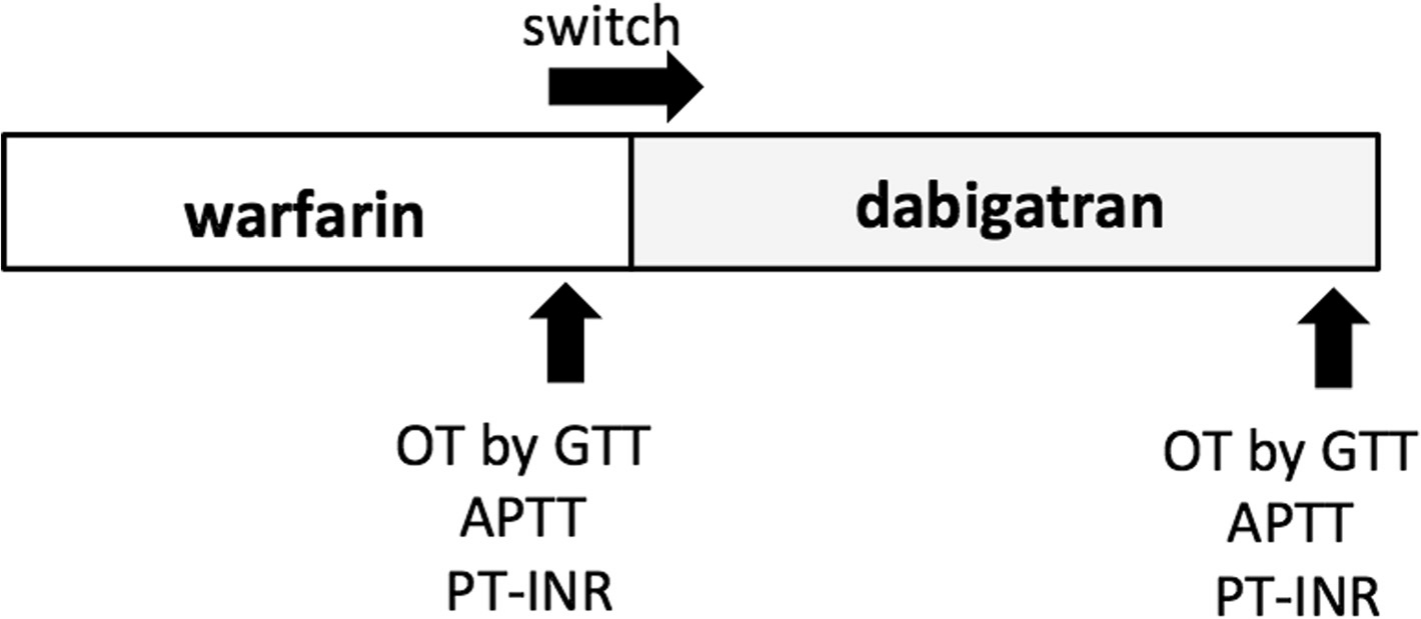
Study protocol: one day before changing the therapy from warfarin to dabigatran and one month after initiating dabigatran therapy, thrombotic status was assessed using the global thrombosis test (GTT), prothrombin time-international normalized ratio (PT-INR), and activated partial thrombin time (APTT) in patients with non-valvular atrial fibrillation. OT, occlusion time

The study population consisted of 50 Japanese volunteers not taking antithrombotic treatment (Male/Female = 27/23, 69.2 ± 1.3 y.o.) as well as 43 Japanese NVAF patients receiving warfarin therapy (Male/Female = 27/16, 76.5 ± 1.4 y.o.). The patients regularly visited at the outpatient clinic of Kobe Rosai Hospital. The value of PT-INR of NVAF patients receiving warfarin was 1.83 ± 0.1. The study protocol is shown in Fig 1 and the clinical profile of subjects is shown in Table 1. The average CHA2DS2-VASc score of NVAF patients was 4.14 ± 0.21, and 60.5 % of patients had a CHA2DS2-VASc score ≥ 4. The average HAS-BLED score was 1.86 ± 0.16, and 32.6 % of patients had a CHA2DS2-VASc score ≥ 3. The thrombotic status of all patients was assessed one day before and 1 month after switching anticoagulation from warfarin to dabigatran (Prazaxa, Boehringer-Ingelheim, Germany) at a dose of 110 mg b.i.d or 150 mg b.i.d, with the GTT, PT-INR and APTT.

**Table 1:** A clinical profile of subjects

Age	76.5 ± 1.4
Male sex, N (%)	27 (62.8 %)
Follow up period (months)	14.7 ± 1.6
CHA_2_DS_2_ –VASc score, N (%)	
0 or 1 point	3 (7.0 %)
2 points	3 (7.0 %)
3 points	11 (25.5 %)
≥ 4 points	26 (60.5 %)
HAS-BLED Score, N (%)	
0 point	4 (9.3 %)
1 point	13 (30.2 %)
2 points	12 (27.9 %)
≥ 3 points	14 (32.6 %)
Hypertension, N (%)	28 (65.1 %)
Diabetes, N (%)	12 (27.9 %)
Dyslipidemia, N (%)	31 (69.8 %)
Concomitantly taking anti-platelets	
Aspirin, N (%)	21 (48.8 %)
Clopidogrel, N (%)	2 (4.7 %)

The present study was approved by the hospital ethics committee, and written informed consent was obtained from all patients.

### Global Thrombosis Test

**Fig. 2:**
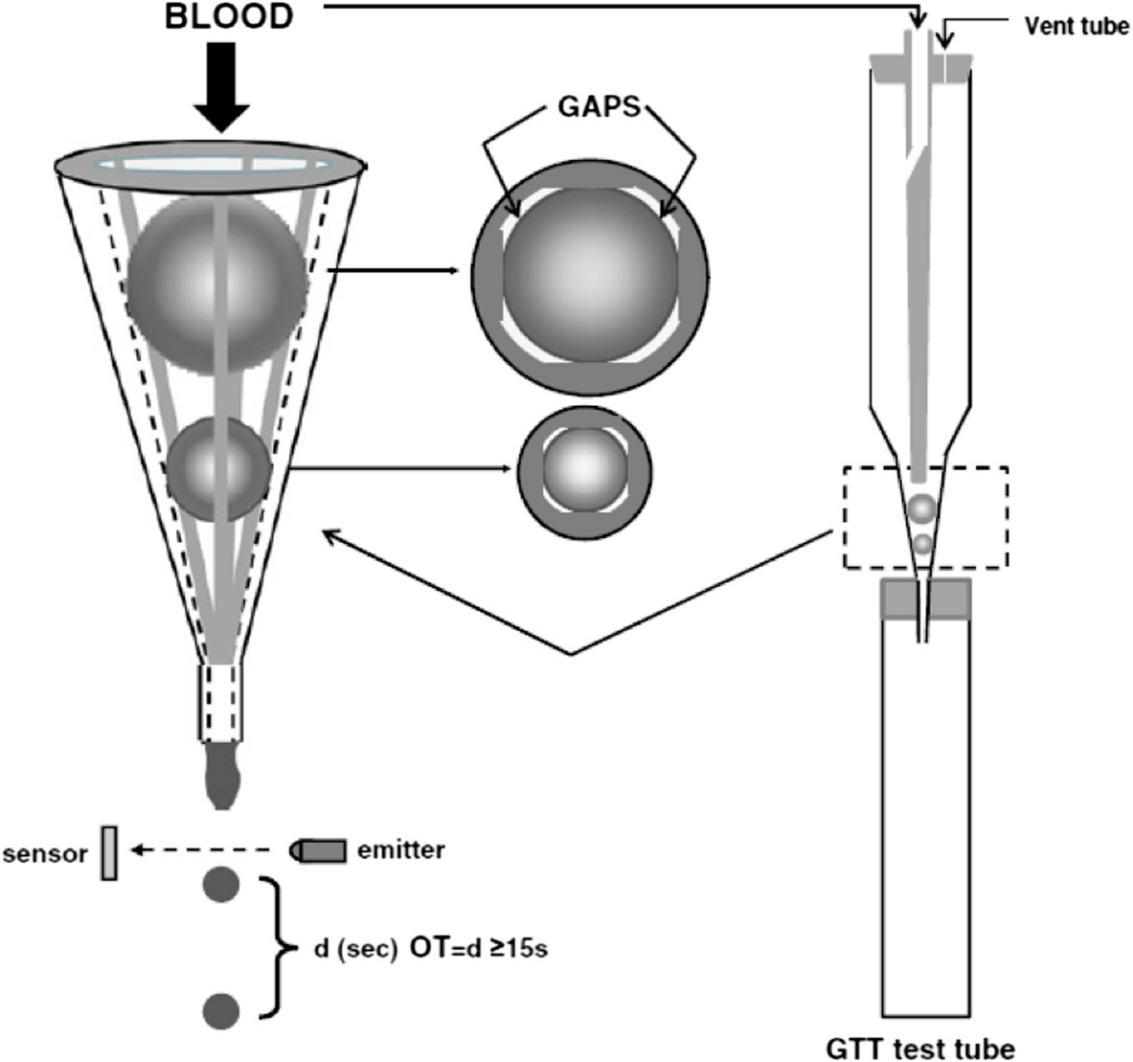
Global thrombosis test (GTT), which is used to measure platelet reactivity, coagulation (thrombin generation), and spontaneous (endogenous) thrombolytic activity

The GTT is a novel, point-of-care assay that employs native (non-anticoagulated) blood. The instrument assesses the time taken to create a shear-induced thrombus under physiological conditions and in the second phase of the test, measures the time to achieve endogenous thrombolysis of the thrombus created during the first phase of the test (Fig 2). The principle of the GTT has previously been described in detail [3, 4]. Briefly, the GTT test tube has a conical part in which two ceramic ball bearings are located. Because of the four flat segments formed on the inner surface of the tube, four narrow gaps exist by the ball bearings. Blood was drawn from the antecubital vein via a 21G infusion set into three 5 ml plastic syringes, taking care to avoid prolonged tourniquet time. When whole blood (4 ml) is added to the GTT disposable tube, it flows through the narrow gaps by the ball bearings and the droplets are collected in a reservoir. Passing through the gaps by the upper (larger) ball bearing, platelets are exposed to high shear stress and activated. In the space between the two balls, platelet aggregates are formed and thrombin is generated from the activated platelets. As the fibrin-stabilized thrombi reach and gradually occlude the gaps by the lower ball bearing, blood flow is reduced and then arrested. The instrument measures the time between 2 consecutive blood drops. This time interval increases gradually as flow slows down. The end point of the measurement is displayed (occlusion time [OT], in seconds). Subsequent to OT, this system can measure endogenous thrombolytic activity by detecting the time taken until restart of blood flow (lysis time [LT], in seconds).

### Follow up and clinical endpoints

Patients were followed up for approximately 18 months for the co-primary endpoints of ischemic events (stroke, non-fatal acute myocardial infarction or cardiovascular death) or major bleeding (classified as type 3–5 according to the Bleeding Academic Research Consortium, BARC definition) [5].

### Statistical analysis

Continuous variables are expressed as medians and inter-quartile ranges. Differences between the two groups were analyzed by the Wilcoxon matched paired test. The correlation between variables was evaluated by Spearman’s rank correlation coefficient. Statistical analyses were performed using IBM SPSS Statistics Version 22. A p-value of <0.05 was considered statistically significant.

## Result and discussion

The non-inferiority of dabigatran compared to warfarin was confirmed by several clinical trials [1] and dabigatran was approved and launched in Japan in late March 2011. However, a considerable number of patients have had severe bleeding, especially among subjects with renal dysfunction [6, 7]. Therefore, there is a compelling need for a monitoring system for dabigatran. The present investigation was undertaken to examine whether the GTT system could be the monitoring system for dabigatran.

**Fig. 3:**
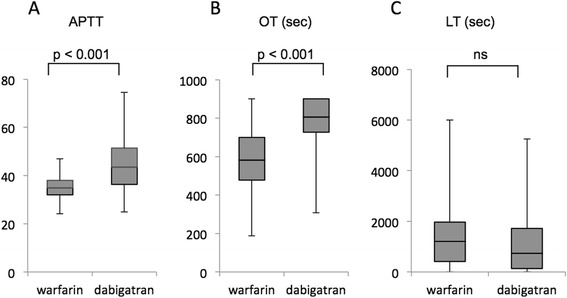
In patients with non-valvular atrial fibrillation, changes in (**a**) activated partial thrombin time (APTT), (**b**) occlusion time (OT), and (**c**) lysis time (LT) following a change from warfarin therapy (left) to dabigatran (right) therapy. The use of dabigatran significantly prolonged APTT as well as OT, compared with warfarin, while there was no change in LT

The OT and LT values assessed by the GTT in normal volunteers without anticoagulants were 524.9 ± 17.0 sec and 1409.8 ± 100.7 sec, respectively. Activated partial thromboplastin time (APTT) and the OT values of NVAF patients under warfarin therapy ranged from 24.1 to 47.0 (mean 35.5 ± 0.7) sec and from 187.9 to 900 (mean 581.7 ± 26.3) sec, respectively. Switching anticoagulation from warfarin to dabigatran resulted in significant prolongation of the APTT values from 35.5 ± 0.7 to 44.8 ± 1.8 sec (Fig 3a). There was also prolongation of the OT values from 581.7 ± 26.3 to 784.5 ± 19.3 sec (Fig 3b). In contrast, there was no significant change in LT in response to the change in anticoagulation (Fig 3c). Therefore, dabigatran appears to exert no additional effect on endogenous thrombolytic activity, over and above that of anticoagulation.

The OT value measured by the GTT system reflects not only platelet reactivity to pathologically relevant high shear stress but also thrombin generation [4]. In other words, the GTT can simultaneously assess both platelet reactivity and the pro-coagulant state. Warfarin inhibits the pro-coagulant state by the suppression of vitamin K-dependent coagulation factors. Since thrombin is a potent activator of platelets, dabigatran, a potent thrombin inhibitor, could affect the platelet activity. Indeed, Eisert WG et al. reported that dabigatran did not inhibit platelet aggregation when induced by arachidonic acid, collagen, or ADP in human platelet rich plasma. However, platelet aggregation activated by thrombin was inhibited by dabigatran [8]. This property could explain our findings that switching anticoagulation from warfarin to dabigatran resulted in the prolongation of the OT.

APTT as well as OT values as assessed by the GTT in NVAF patients were significantly increased by switching anticoagulation from warfarin to dabigatran. These findings suggest the possibility that GTT could be the monitoring system for NOAC including dabigatran. Recently, Suzuki et al. demonstrated a wide distribution of APTT values in Japanese NVAF patients receiving dabigatran therapy, and they proposed that the monitoring by APTT might aid in the screening of patients at increased bleeding risk with dabigatran [9]. We examined the correlation between the OT value and APTT or PT-INR. Spearman’s rank correlation coefficient and p- values between OT value and APTT were 0.245 and 0.114, respectively. Spearman’s rank correlation coefficient and p- values between OT value and PT-INR were 0.178 and 0.254, respectively. Thus, there was no significant correlation between OT and APTT or PT-INR. Therefore, the OT value evaluated by the GTT is independent of APTT or PT-INR.

At elevated shear stress such as that which occurs in a stenosed coronary artery, platelet thrombus formation depends entirely on von Willebrand factor (vWF) binding to platelet glycoproteins Ib/IX/V and IIb/IIIa. Shear-induced platelet activation and aggregation is initiated by the plasma vWF interacting with the platelet GPIb/IX/V complex and platelet crosslinking through integrin-aIIbβ3. In addition to its role in platelet activation and aggregation, vWF has an important role in physiological hemostasis, in the formation of platelet plugs at sites of endothelial damage. VWF binds to the exposed subendothelium and forms a bridge between this surface and to the platelets, where it binds to glycoprotein complexes Ib/IX/V and IIb/IIIa, which act as vWF receptors. Inherited deficiencies or dysfunctions of vWF cause a bleeding disorders and vWF level correlates inversely with bleeding risk [10]. Earlier published research evaluating the mechanism of the thrombotic occlusion in the GTT has revealed the fundamental role of vWF in determining the OT value. Antagonism of vWF with aurin tricarboxylic acid (ATA) significantly inhibited OT in the GTT [11]. Furthermore, platelet-rich thrombus formation in the GTT was dose-dependently inhibited by monoclonal antibody against platelet glycoprotein (GP) Ib. The OT value was inversely correlated with vWF ristocetin cofactor activity and vWF antigen. This report is the first to clearly demonstrate the role of vWF in the formation of occlusive thrombi in the Gorog Thrombosis Test [12]. Thus, the OT value in the GTT system is directly determined by vWF. Although our present study was not powered to assess the predictive value of prolonged OT on the risk/propensity for bleeding, intuitively, since vWF is a sensitive marker of bleeding risk, it seems reasonable to suppose that extreme prolongation of OT will be related to increased bleeding risk. In the present investigation, the number of subjects with OT > 900 sec on warfarin therapy was one among 43 NVAF patients, whereas with dabigatran therapy there were 12 patients with OT > 900 sec. There were no ischemic events and no major bleeds observed amongst the patients during the follow up period of (14.7 ± 1.6 months). Further assessment of this in larger populations followed up for a longer period is necessary to better evaluate the usefulness of OT, as assessed by the GTT as a monitoring system to assess bleeding risk with NOAC.

Antithrombotic therapy for the prevention of cardiovascular disease and ischemic stroke is inevitably associated with bleeding risk; therefore, the most crucial thing is to reconcile the two conflicting aspects, that is, the anti-thrombotic effects and the bleeding risk. To this end, it would be highly desirable to establish clinical tools for estimation of coagulant and thrombotic status. The incidence of NVAF increases with age; therefore, concomitant atherosclerosis-based cardiovascular diseases such as angina pectoris and myocardial infarction also occur more frequently. In such cases, the combination of anticoagulation and antiplatelet therapy is necessary, but the bleeding risk is inevitably increased with combination therapy. A comprehensive assessment is important for assessment of the thrombotic risk, as well as bleeding, in patients treated with antiplatelet therapy and anticoagulation. Since the GTT can simultaneously assess both platelet reactivity and the pro-coagulant state, this system seems to have significant potential for the assessment of ischemic risk in various cardiovascular disease states. Furthermore, the GTT system has several advantages over previous methods. The GTT does not require anti-coagulants for the measurement, and the results are unaffected by the procedure of sample preparations. Right now a large scale of clinical trial using the GTT system is under progression.
